# Prevascular Richter's hernia a rare and challenging presentation of a potentially life-threatening condition: A rare case report

**DOI:** 10.1016/j.ijscr.2024.110024

**Published:** 2024-07-10

**Authors:** Mohammad Al-Jawad, Sidra Alnasser, Nour Abdulazize Lbabidi, Alan Sheikh, Bashar Diarbakerly, Abdulmonem Kawas

**Affiliations:** University of Aleppo, Faculty of Medicine, Aleppo, Syria

**Keywords:** Richter's hernia, Prevascular, Richter type B

## Abstract

**Introduction:**

Richter's hernia is a relatively uncommon type of hernia that can lead to severe clinical consequences if left unaddressed. The definitive treatment involves the reduction and repair of the hernia defect, with various surgical approaches available, including open transabdominal, inguinal, obturator, and laparoscopic techniques, depending on the size of the defect and the viability of the involved bowel.

**Case presentation:**

A 29-year-old female patient presented with Richter's hernia, a rare type of hernia, and underwent surgical intervention to release the incarcerated bowel loop and resect the necrotic segment. The case emphasizes the need to consider Richter's hernia in atypical hernia presentations to prevent severe complications.

**Discussion:**

Richter's hernia is a distinct type of hernia characterized by the entrapment of a portion of the intestinal circumference, often leading to rapid onset of gangrene without causing intestinal obstruction. The diverse anatomical locations of Richter's hernias, including the rare prevascular variant, emphasize the importance of maintaining a high index of suspicion for this condition, particularly in the context of laparoscopic interventions and atypical hernia presentations.

**Conclusion:**

Early recognition and prompt referral for appropriate imaging investigations are essential to prevent the significant morbidity and mortality associated with this notoriously challenging-to-diagnose condition, and a heightened awareness and a comprehensive diagnostic approach are crucial to effectively identify and address this rare but potentially life-threatening surgical emergency.

## Highlights

• Richter's hernia is a rare but severe hernia type requiring prompt surgical repair.
• Surgical approaches include open and laparoscopic techniques to reduce and repair the defect.
• Early diagnosis through imaging is crucial to prevent the high morbidity and mortality.

## 1 Introduction

Diverticulosis coli is a prevalent gastrointestinal disorder, but its underlying pathogenesis remains poorly understood. Various risk factors have been implicated, including gender, genetics, neuromuscular abnormalities, mucosal inflammation, diet, and obesity, all of which may contribute to the development and progression of this condition, though the precise mechanisms are not yet fully elucidate (1).

Richter's hernia, a type of condition associated with diverticulosis coli, is a relatively uncommon entity within the hernia family, yet it can lead to severe clinical consequences if left unaddressed. This type of hernia involves the herniation of the anti-mesenteric portion of the bowel through a fascial defect, often resulting in subtle or subclinical symptoms that can delay presentation. The increasing prevalence of minimally invasive surgeries has contributed to a rise in the incidence of Richter's hernias (2).

The definitive treatment involves the reduction and repair of the hernia defect, with various surgical approaches available, including open transabdominal, inguinal, obturator, and more recently, laparoscopic techniques (3).

The choice of repair method typically depends on the size of the defect and the viability of the involved bowel, which may necessitate bowel resection in addition to the hernia repair. While primary suture closure may be suitable for small defects, the trend has shifted towards the use of polypropylene mesh due to its strength, non-absorbable nature, and potential resistance to infection, particularly in the context of larger or more complex Richter's hernia defects (2,3).

We report the case of a 29-year-old female patient who was found to have a rare prevascular-type Richter's hernia. Richter's hernias are an uncommon form of this surgical condition, and the prevascular variant is an exceptionally infrequent presentation. This work has been reported in line with the SCARE 2023 criteria (4).

## 2 Case presentation

We report the case of a 29-year-old female patient who presented to the emergency department with a 3-day history of cramping periumbilical pain that had progressively worsened and spread to the entire abdomen. The patient also reported severe bilious vomiting that occurred frequently after meals, accompanied by intolerance to oral intake. Additionally, the patient complained of constipation and urinary discomfort.

On physical examination, a tender swelling was noted in the inguinal region. The abdomen was soft with normal respiratory excursions, and the patient's vital signs were notable for relative hypotension (90/60 mmHg). Laboratory studies demonstrated a normal complete blood count, mild hyponatremia (sodium 131 mEq/L), hypokalemia (potassium 3.36 mEq/L), and elevated inflammatory markers (C-reactive protein 237 mg/L). Renal function tests revealed an elevated urea level of 101 mg/dL and a mildly elevated creatinine of 0.6 mg/dL, which were consistent with pre-renal azotemia, likely secondary to dehydration, rather than primary renal injury.

**Fig. 1:**
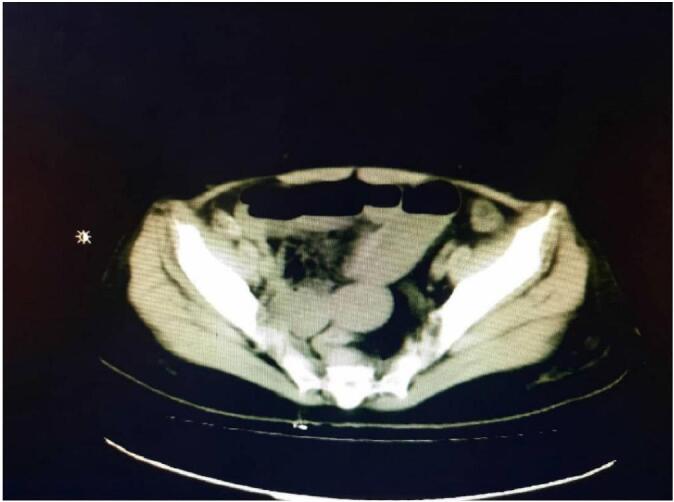
A CT scan image showing Dilated intestinal loops with aerobic fluid levels.

Radiologic imaging, including contrast-enhanced computed tomography, showed dilated small bowel loops with intraluminal air-fluid levels, suggestive of intestinal obstruction (Fig. 1). An ultrasound of the groin confirmed the presence of an incarcerated intestinal loop within a hernia sac.

**Fig. 2:**
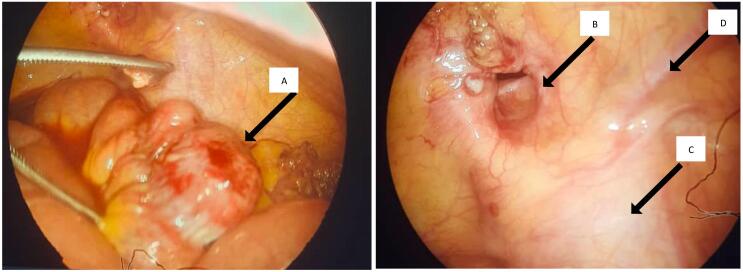
The edematous, herniated loop in the ostium of the hernia neck and Femoral vessels in front of the herniated loop that indicates type B femoral hernia.A.Intestinal hernia within the contents of the hernia sac.B.prevascular-type hernia (Type B)C.iliac vesselsD.Epigastric vessels

Initially, it was thought to be an indirect inguinal hernia (Type A) that passed lateral to the epigastric vessels. However, after dissection during laparoscopic surgery, it was confirmed that the loop had passed through the femoral canal, and the hernia was thus classified as a Richter-type femoral hernia (Type B) (Fig. 2), which has a different anatomical location, passing in front of the femoral vessels.

As the hernia was located approximately 100 cm from the ileocecal junction, the incarcerated intestinal loop was released through an endoscopic approach. However, the surrounding peritoneum failed to be adequately released, prompting a conversion to open surgical management using the Lotheissen technique to avoid potential femoral vascular damage.

An incision was made parallel and 2 cm above the inguinal ligament, exposing the round ligament and femoral ring. A mesh plug was placed and fixed, the necrotic omentum was excised, and the free abdominal fluid was aspirated. The patient's postoperative course was uncomplicated, and she remained symptom-free on follow-up.

## 3 Discussion

Richter's hernia, first reported by Fabricius Hildanus in 1598 and later scientifically described by August Gottlob Richter in 1778, is a distinct type of hernia characterized by the entrapment of a portion of the intestinal circumference and strangulation at the hernia orifice, often leading to rapid onset of gangrene without necessarily causing intestinal obstruction. Approximately 5–15 % of strangulated hernias are Richter's hernias, with an increasing incidence at sites of laparoscopic port insertion. Typically, Richter's hernia involves the distal ileum, and a tight constricting ring is a prerequisite for strangulation. While the most common sites of involvement are femoral and inguinal, spontaneous Richter's hernia can occur through other defects in the abdominal wall (5).

Richter's hernias can manifest in diverse anatomical locations, including the inguinal, incisional, umbilical, and Spigelian regions. Additionally, a less common variant known as the prevascular type can occur, where the hernia protrudes through a defect in the abdominal wall in front of the major blood vessels. Furthermore, with the growing adoption of laparoscopic surgical techniques, clinicians have reported an increasing number of Richter's hernias occurring at the port sites used for these minimally invasive procedures.

While the distal portion of the small intestine is the gastrointestinal segment most frequently involved in Richter's hernias, other regions of the digestive tract may also be affected. This variability in the specific bowel segment compromised underscores the need for clinicians to maintain a high index of suspicion for this condition, as it can present with a diverse range of anatomical manifestations.

The diverse anatomical locations, including the prevascular variant, and the potential involvement of various gastrointestinal structures emphasize the importance of healthcare providers remaining vigilant and considering Richter's hernia as a possible diagnosis, particularly in the context of laparoscopic interventions and atypical hernia presentations (6).

**Fig. 3:**
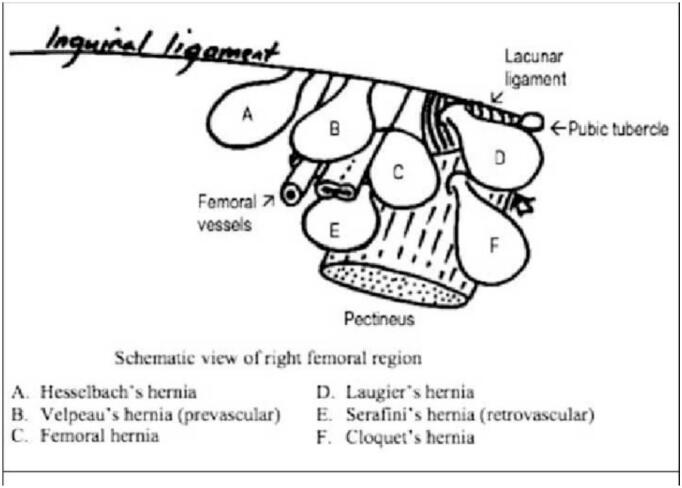
A diagram showing the different types of Richter's hernia.

The patient in the present case was found to have a prevascular-type hernia, a variant that is exceedingly uncommon in clinical practice (Fig. 3).

Patients with a Richter's hernia, including the rare prevascular variant, often present with similar symptoms to those seen in other types of incarcerated hernias, such as abdominal discomfort, distension, nausea, and vomiting. However, a key distinguishing feature is the delayed onset of these obstructive symptoms. This is due to the fact that Richter's hernias involve only a portion of the bowel wall, without complete obstruction of the intestinal lumen. As a result, patients may initially experience subclinical symptoms until the condition progresses, leading to strangulation of the affected bowel and a subsequent exacerbation of the clinical presentation. When evaluating these patients, it is crucial to obtain a comprehensive medical history, with particular attention to predisposing factors like prior hernias or a history of minimally invasive surgery. Additionally, clinicians should carefully assess any comorbidities, active cardiopulmonary disease, and use of anticoagulation, as these factors may necessitate specific preoperative management considerations if surgical intervention is required (3).

The definitive treatment for Richter's hernias involves the reduction of the hernia contents and subsequent repair of the underlying defect in the abdominal wall. A variety of surgical approaches have been described to address this condition, including open techniques through the abdomen, groin, or obturator foramen, as well as minimally invasive laparoscopic procedures, both extraperitoneal and transperitoneal. The choice of approach may depend on the specific location and characteristics of the hernia.

In terms of the hernia defect closure, smaller defects may be amenable to primary suture repair, while larger or more challenging defects may require the utilization of native tissue reinforcement, such as the omentum, a peritoneal patch, or a pectineal muscle flap. Furthermore, the incorporation of prosthetic mesh has become a popular trend in the repair of Richter's hernias, as these synthetic materials offer the advantages of increased strength, non-absorbability, and potential resistance to infection.

The selection of the most appropriate surgical technique and closure method should be tailored to the individual patient's anatomy, hernia characteristics, and clinical context, to achieve a durable and definitive repair while minimizing the risk of recurrence or complications (7).

In our case, the patient presented with the chief complaint of generalized abdominal pain and repeated episodes of green-colored vomiting, which prompted the hospital visit. The patient was managed surgically, with the release of the incarcerated bowel loop and resection of the necrotic segment to address the complications of the Richter's hernia.

## 4 Conclusion

Early recognition and prompt referral for appropriate imaging investigations are essential to prevent the significant morbidity and mortality associated with this notoriously challenging-to-diagnose condition. The prevascular type of Richter's hernia, though extremely infrequent, should not be overlooked and should be included in the differential diagnosis, especially in cases with atypical or ambiguous presentations.

Timely and accurate diagnosis, followed by appropriate interventions, is paramount in managing Richter's hernias, regardless of their specific anatomical manifestation, to optimize patient outcomes and minimize the risks of complications. A heightened awareness and a comprehensive diagnostic approach are essential to effectively identify and address this rare but potentially life-threatening surgical emergency.

## Informed consent

Unnecessary, information taken from the patient's file.

## Consent for publication

All authors provide consent for publication.

## Provenance and peer review

Not commissioned, externally peer-reviewed.

## Ethical approval

Not applicable.

## Funding

There are no funding sources.

## Author contribution

The work's conception and design: all authors.

paper writing, and article revision: all authors.

Final revision and approval: all authors.

## Guarantor

Abdulmonem Kawas.

## Research registration number

Our research study does not involve human subjects.

## Conflict of interest statement

The authors declare that they have no competing interests.
